# Supplementary material for: Genetic Diversity and Geographical Distribution of the Red Tide Species Coscinodiscus granii Revealed Using a High-Resolution Molecular Marker
Source: Microorganisms. 2022 Oct 14;10(10):2028. doi: 10.3390/microorganisms10102028 (PMC9612147; doi:10.3390/microorganisms10102028)
Supplement: Supplementary file 1 [file microorganisms-10-02028-s001.zip › Figure S1.pdf]

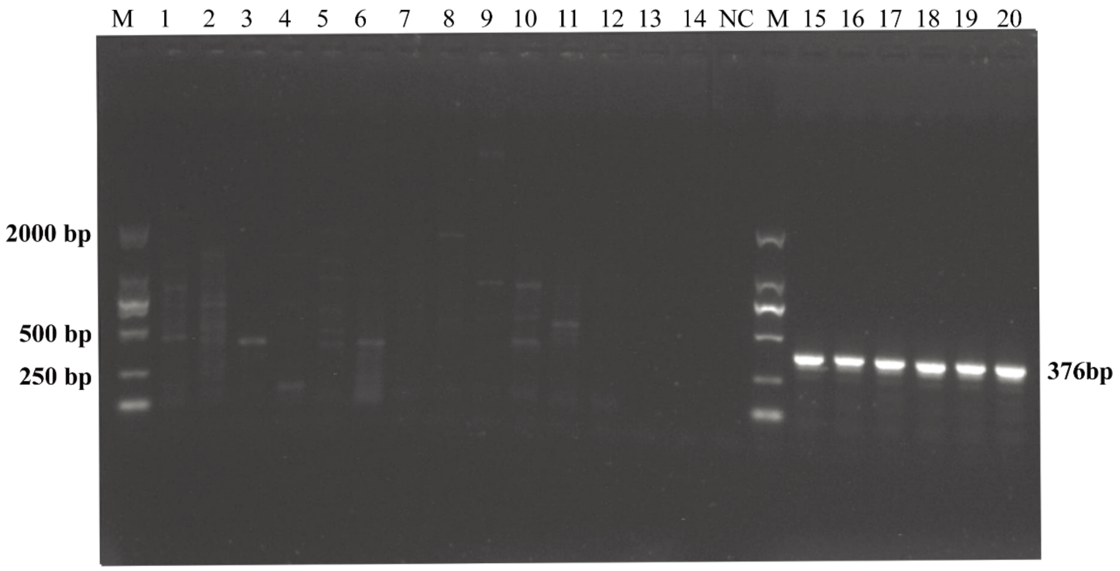

Figure S1 Specificity of molecular marker *cgmt1*. M: DL2000 marker; Lanes1-14: *Heterosigma akashiwo*; *Amphidinium carterae*; *Isochrysis galbana*; *Skeletonema costatum*; *Thalassiosira weissflogii*; *Alexandrium tamarense*; *Karenia mikimotoi*; *Prorocentrum donghaiense*; *Aureococcus anophagefferens*; *Chaetoceros curvisetus*; *Chattonella marina*; *Phaeocystis globosa*; *Coscinodiscus wailesii*; *Coscinodiscus* sp.; Lanes15-20: six *C. granii* strains (CNS00613; CNS00614; CNS00749; CNS00746; CNS00554; CNS00741); NC: (double-distilled water) negative control without the addition of DNA template.
