# Supplementary material for: Genetic Diversity and Geographical Distribution of the Red Tide Species Coscinodiscus granii Revealed Using a High-Resolution Molecular Marker
Source: Microorganisms. 2022 Oct 14;10(10):2028. doi: 10.3390/microorganisms10102028 (PMC9612147; doi:10.3390/microorganisms10102028)
Supplement: Supplementary file 1 [file microorganisms-10-02028-s001.zip › Table S1.pdf]

Table S1 The sampling station information of environmental samples

Longitude (°E)	Latitude (°N)	Stations	Sampling time
110.07	20.2	ZN1-7	2019.01
109	21.17	ZN4-3	2019.01
109.33	21.17	ZN4-4	2019.01
109.01	20.17	ZN1-3	2019.01
108.59	21.67	ZN6-2	2019.01
108.61	20.5	ZN2-2	2019.01
120.38	36.03	D8	2019.03
120.29	36.02	D5	2019.03
120.23	36.04	D3	2019.03
120.23	36.07	D1	2019.03
120.29	36.1	C4	2019.03
120.25	36.1	C3	2019.03
120.18	36.1	C1	2019.03
120.19	36.13	B2	2019.03
120.33	36.15	A5	2019.03
120.25	36.16	A3	2019.03
121.23	31.66	C2	2019.03
121.91	31.15	C8	2019.03
123	30.72	A6-7	2019.03
122.25	30.95	A6-2	2019.03
121.78	31.41	B4	2019.03
121.49	31.5	B2	2019.03
122.57	29.72	A10-2	2019.03
122.84	29.62	A10-4	2019.03
123.48	29.98	A8-6	2019.03
122.7	30.23	A8-2	2019.03
117.7	22.73	101-2	2019.06
116.95	22.64	Mar-96	2019.06
115.99	22.44	Mar-84	2019.06
115.52	22.17	Jan-75	2019.06
111.5	19.59	125-1	2019.06
111.29	20	118-1	2019.06
114.34	22.09	Feb-46	2019.06
113.93	21.45	Feb-41	2019.06
113.31	21.3	2 月 27 日	2019.06
112.84	21.51	3 月 16 日	2019.06
121.67	26.6	S05-3	2019.09
121.99	26.4	S05-4	2019.09
123.32	26.76	S04-5	2019.09
122.06	27.54	S04-2	2019.09
122.7	28.37	S03-2	2019.09
123.09	28.15	S03-3	2019.09

123.22	29.38	S02-2	2019.09
122.8	29.6	S02-1	2019.09
124.8	30	S01-5	2019.09
124.35	30.25	S01-4	2019.09
121.17	37.93	N24	2019.10
121.27	38.29	N22	2019.10
122.11	38.65	N18	2019.10
122.25	37.76	N14	2019.10
122.92	38.39	N12	2019.10
123.25	38.76	N10	2019.10
123.77	39.22	N08	2019.10
123.74	38.23	N06	2019.10
123.24	37.74	N04	2019.10
123.51	37.5	N02	2019.10
119.32	38.47	B25	2019.10
119.67	38.87	B23	2019.10
120.24	39.33	B21	2019.10
120.92	39.54	B19	2019.10
120.32	39.13	B15	2019.10
119.71	39.31	B13	2019.10
118.97	38.94	B11	2019.10
118.97	38.65	B09	2019.10
119.44	38.35	B05	2019.10
120.21	38.34	B03	2019.10
